# A Qualitative Exploration of Perceptions and Experiences of Adolescent Girls and Young Women on Modern Contraceptive Methods Use in Malinyi District, Morogoro, Tanzania

**DOI:** 10.24248/eahrj.v8i3.806

**Published:** 2025-01-30

**Authors:** Ashura S Mkande, Idda H. Mosha

**Affiliations:** a School of Public Health and Social Sciences, Muhimbili University of Health and Allied Sciences, Dar es Salaam, Tanzania; b Department of Behaviour Sciences, School of Public Health and Social Sciences, Muhimbili University of Health and Allied Sciences, Dar es Salaam, Tanzania.

## Abstract

**Background:**

Despite the existence of plans and strategies for providing family planning methods in Tanzania, the uptake rate of modern contraceptives among Adolescent Girls and Young Women (AGYW) in Tanzania remains low. The fertility rate is 112 per 1000; only 15.2% of adolescents are using Modern Contraceptives Methods (MCMs) in Tanzania. Modern contraceptive use is one of the important interventions to reduce the burden of adolescent pregnancy which is as high as 22% in the country. However, little is known regarding AGYW’s perceptions and experiences with MCMs.

**Objective:**

To explore the perceptions and experiences of AGYW with MCMs use in the Malinyi district of Morogoro, Tanzania.

**Materials and Methods:**

This was a qualitative phenomenology study. Purposive sampling was used to select 19 study participants. An in-depth interview guide was used to collect data. Data were transcribed verbatim. A thematic data analysis approach was used to analyze data.

**Results:**

Two major themes that emerged from the findings were experiences and perceptions of AGYW on MCM use. On the experiences of MCMs, the study findings revealed that AGYW have limited knowledge and awareness about modern contraceptive methods; some of them acknowledged the benefits of modern contraceptive methods in preventing unintended pregnancies, providing a sense of empowerment, and enabling them to pursue their life goals. On perceptions of MCM use some of the study participants shared challenges encountered, including side effects, social influence, cultural and religious beliefs, myths and misconceptions that contribute to the perceptions and use of MCMs.

**Conclusion and recommendation:**

Healthcare providers at the health facilities should continue educating AGYW on the importance of using MCMs.

## BACKGROUND

World Health Organization (WHO) defines ‘Adolescents’ as individuals in the 10 to 19 years age group and ‘Youth’ as the 15 to 24 year age group. While ‘Young People’ covers the age range 10 to 24 years ^1^. Globally, adolescents continue to face substantial unmet Sexual and Reproductive Health (SRH) needs. In 2017, it was estimated that 36 million girls aged between 15 and 19 years were either married or sexually active but did not wish to become pregnant in the next two years. Of these girls, over 20 million needed a Modern Contraceptive Method (MCM), yet they were not using one.^2^

Globally, only 12% of girls aged between 15 and 24 years use modern contraceptive methods.^3^ Sub-Saharan Africa recorded the lowest use of MCMs.^4^ In 2019, only 17.9% of women aged 15–19 years were using modern contraceptive methods.^5^ Similarly, in East Africa, there is low usage of MCMs among adolescents.^6^ In Uganda, only 9.4% of female adolescents use MCMs.^4^ However, in Kenya, 42.9% of adolescents use MCMs.^32^ Adolescents exercise independence by engaging in risk-taking behaviours such as substance abuse, alcohol intake, and unprotected sexual intercourse.^3^

Sexual intercourse puts adolescents at risk of contracting sexually transmitted diseases and early pregnancies. Hence, adolescent sexual reproductive health has been emphasised at global and local levels.^3^ The World Health Organization (WHO) recommends governments to take steps to ensure the provision of comprehensive reproductive health education, counselling, and provision of contraceptives.^5^ At the local level, Tanzania has established various strategies and plans to improve adolescents’ reproductive health. For instance, the National Adolescent Reproductive Health Strategy (2011-2015)^7^ and the National Road Map Strategic Plan for Reproductive, Maternal, Newborn, Child and Adolescent Health in Tanzania (2016-2020),^8^ have been established to address the reproductive health needs of adolescents. Such needs include reproductive health advice, Sexually Transmitted Diseases (STDs) testing, post-abortion, postnatal, and childbirth care, contraception and condom use promotion and provision.^8,9^ Despite the existence of plans and strategies to enhance contraceptive use, the rate of use of modern contraceptives among adolescent girls and young women (AGYW) remains low in the Morogoro region specifically in the Malinyi District.

While awareness of modern contraceptive methods among AGYW (15–24 years) is high with 96% being aware, only 15.2% use such methods.^4,10^ In Tanzania, 96% of adolescents are aware of modern contraceptives but only 15.2% use any of these methods.^10^ Low use of MCMs among girls aged 15 to 19 years is estimated at 11% of births globally occur among girls aged 15 to 19 years, 95% of which appear in low- and middle-income countries (LMICs).^11^ It has been revealed that the overall collective prevalence of pregnancy among adolescents is 19.3% in sub-Saharan Africa (SSA) and that 120 out of every 1000 girls aged 15–19 years within SSA experience unplanned pregnancies. This prevalence is highest in the East African region at 21.5%.^12^

Worldwide it is estimated that at the age of 17, adolescent girls are 5 times more likely to conceive if they do not use contraceptives compared to those who use contraceptives at their first sexual intercourse.^13^ In Tanzania, low intake of contraceptives among AGYW has resulted in teenage pregnancies. Tanzania has one of the highest adolescent pregnancy rates being ranked the 17^th^ in Africa where teenage pregnancy in the country has been increasing from 23% in 2010 to 27% in 2015.^5^ Additionally, Tanzania has a high teenage pregnancy rate with at least 6% of teenage girls aged 15 to 19 years currently being pregnant.^14^ Besides, the current Modern Contraceptive Prevalence Rate (m-CPR) among adolescents is 15.2%. This shows a significant decline from 18.9% in 2015 and 2016 which is greatly lower compared to the national average of 32%.^15^ This uncovers that unmet need for MCMs is high among adolescents aged 15 to 19 years in Tanzania and there is little progress in rates of unmet need for modern contraception use (30% in 2004/05, 25.3% in 2010 and 26.5% in 2015/16 respectively.^14^

The challenge of teenage pregnancies resulting from non-frequent use of contraceptive methods affects school girls in Tanzania. At least 6,500 girls drop out of school because of pregnancy every year.^16^ For example a study by Ngilangwa et al,^16^ in Tanzania on the experiences of contraceptive use among AGYW, revealed that their close relations significantly influenced contraceptive use and decision-making on using modern contraceptives. Likewise, it was revealed that schools and healthcare play a role in shaping attitudes and fostering motivation for contraceptive use or misuse.^16^ MCMs can prevent adolescents from conceiving and thus prevent negative health consequences to adolescent mothers and their babies, as well as socio-economic consequences such as stigma and violence.^17^

Despite the availability of modern contraceptive methods, AGYW continues to have low contraceptive use rates due to factors such as lack of awareness, access to appropriate contraceptives, social norms stigma, and myths surrounding modern contraceptive methods.^15^ The use of MCMs among AGYW in rural areas is limited and young women are affected by not using MCMs. AGYW’s perceptions and experiences on the use of MCMs become a challenge^16^ accompanied by risky behaviours such as unprotected sexual intercourse and unplanned pregnancies.^2^ Although statistics show that 15% of AGYW in Tanzania use MCMs, the contraceptive prevalence in Morogoro is relatively high at 49% among women aged 15 to 49. However, the pregnancy rate among adolescents is so high at 39%^5,4^ with Malinyi district experiencing a particularly higher rate of adolescent pregnancy at 48.6%.^18^ This might suggest that MCMs use among AGYW is low. Low uptake of MCMs might be caused by perceptions and experiences AGYW have on these methods.^3^ Therefore, from this backdrop, this study aimed to explore the perceptions and experiences of AGYW regarding MCMs use in rural settings, particularly in the Malinyi district of Morogoro, Tanzania.

## MATERIALS AND METHODS

### Study Design and Setting

This was a phenomenological qualitative study design. An in-depth interview (IDI) guide was used to explore perceptions and experiences of modern contraceptive use among AGYW in the Malinyi District Morogoro, Tanzania.

### Study Area

The study was conducted in the Malinyi district in Morogoro Region. The district is one of the nine (09) Councils of the Morogoro Region with a total area coverage of 9979.9 Km^2^. The Malinyi District is located about 370 kilometres from Morogoro Regional Headquarters in the southern part of Morogoro Region. It borders Namtumbo District (Ruvuma region) and Njombe District to the south, Ulanga and Liwale Districts to the east and Kilombero District to the northwest. Compared to the other seven districts Malinyi district has the highest prevalence rate of teenage pregnancies, standing at 49.2% according to DHIS2 (2020),^20^ followed by Ifakara town with 35.9%. Despite the region having a high contraceptive prevalence rate, teenage pregnancies remain a significant issue in the Morogoro Region which is among the top three Regions with 39%.^21^ Data were collected in May 2023.

### Study Population

A purposive sampling was used to select AGYW aged 15–24 years who are using or had previously used MCMs. The AGYW who were using or had previously used MCMs were included in this study because they could explain their experiences and perceptions regarding the use of MCMs.

### Sample Size and Sampling

A purposive sampling was used to select AGYW (15–24 years) who were using or had previously used MCMs. This sampling technique was used as it enables researchers to select participants based on specific characteristics, experiences, or perspectives relevant to the research topic, ensuring a deeper understanding of the phenomenon under investigation.^22^ Data were collected at the community level. The District Reproductive and Child Health Coordinator assisted in selecting two wards served by the facilities with family planning services. The selected wards had a huge numbers of AGYW who are served family planning services by the facilities. In the first stage for the selected wards, the Ward Executive Officer (WEO) was informed about the study. Subsequently, the WEO informed village leaders about the study. The identification of AGYW was done through community health workers who were working at the community level from the selected wards who were visited at their homes or places of work. The participants included in this study were AGYW who were currently using modern contraceptive methods or who had previously used contraceptive methods. The sample size of 19 participants was recruited in the study after reaching saturation, a point at which no new information emerged from subsequent interviews.^22,23^

### Data Collection Method

This study employed an in-depth interview (IDI) as a data collection method. As the use of MCMs is a sensitive issue, IDI method helped in maintaining confidentiality, provided flexibility, captured individual experiences and allowed exploration of the perceptions of AGYW on MCMs use.^24,25^ Data were collected by the principal researcher (PI) assisted by two research assistants with degrees in Social Sciences. The questions in the in-depth interview guide covered demographic characteristics, experiences on modern contraceptive methods use and the perceptions of modern contraceptive methods among the AGYW. The interview guide was developed in English and translated into Kiswahili language as the primary language of communication. Before data collection, a pretest of the data collection tool was conducted in the Biro ward in the Malinyi district to check the clarity of questions in the interview guide. A recording device and written notes were employed to capture data during interviews, which lasted for an average of 60 minutes each. Interviews were conducted in the Swahili language. Conducive places were secured to provide privacy and free conversation between the PI, research assistants and the study participants. Two IDIs were executed each day. This facilitated reflection on, and consolidation of, emerging issues for further questioning.

### Data Analysis

Data were transcribed verbatim. The data underwent a thematic analysis by applying five stages according to Braun and Clarke to establish meaningful patterns: familiarization with the data, generating initial codes, searching for themes among codes, reviewing themes and presenting the results.^26,27^ Nvivo 12 version computer software was used as an aid in the data analysis process. The presented findings capture the essence of the data with direct quotes from the participants.^28^

### Ethical Consideration

Ethical clearance to conduct this study was obtained from the Muhimbili University of Health and Allied Sciences Institutional Review Board (Ref Number DA.282/298/01.C/1732). Furthermore, data collection permissions were sought from the Morogoro Regional office, Malinyi District Council and then Ward level authorities. Informed written consent was obtained from all study participants to confirm their willingness to participate after they had received an explanation of the objectives. Those who were below 18 and were willing to participate consent was sought from the parents or guardian. Permission to use a digital recorder was sought from the study participants before they were interviewed. To ensure privacy and confidentiality of the participants no names were recorded during the interviews. Participants’ voluntary participation and right to withdraw from the study at any time were emphasized.

## RESULTS

### Demographic Characteristics

A total of nineteen 19 participants aged 15 to 24 years who had used MCMs for at least one year and above and stayed in Malinyi for one year and above were included in this study. Most of them were young women between the ages of 21 and 24 years. Out of these, 2 were below 18 years (2 were adolescents) of age, 7 were aged 18 to 20 (6 were adolescents while 1 was a young woman), and 10 were between 21 to 24 years old (10 were young women). Out of 19 participants 8 were adolescents while 11 were young women. Regarding education level nine study participants had completed secondary education, while six had primary-level education, 3 had attended college, and 1 had not attended formal schooling.

About marital status, 12 participants were in a relationship or dating, 4 were married, and 3 were currently single. Among all the study participants, 7 were currently using MCMs, while 12 had used them in the past but they no longer did.

From data analysis, two main themes emerged, which are: experiences and perceptions of MCMs use among AGYW in the Malinyi District. The themes on experiences of the MCMs have the following sub-themes: knowledge and awareness, prevention of unintended pregnancies, empowerment and control of reproductive choices, MCMs use and sexual relationships and challenges and side effects. The perceptions of MCMs theme have the following sub-themes: social stigma, myths and misconceptions and cultural and religious beliefs.

### Theme 1: Experiences of using MCMs

#### Knowledge and Awareness

Study participants were asked about their knowledge and awareness regarding the use of MCMs. Many of the study participants reported that they understood the importance and benefits of the MCMs. However, some of the participants reported the fact that most of the AGYW in the Malinyi district are unaware of the MCMs and the benefits of using them. They further recounted that they did not know what to do when they suffered from side effects. They continued lamenting that the side effects MCMs made many AGYW not to use them. For example, one participant said;

*I think the level of knowledge and awareness on the MCMs is still low among adolescents; most of us do not have enough information regarding these methods, especially on the benefits and the side effects and what to do when suffering from the side effects caused by using the MCMs and that cause many not to use them*. (IDI, PT-02, 2023)

On the other hand, a few study participants reported Experiences of Adolescent Girls on Use of Modern Contraceptive Methods www.eahealth.org that some AGYW in other areas have some knowledge and awareness regarding the use of MCM. They narrated that in some urban areas, some youths understand the importance and benefits of using MCMs. They further recounted that more information is needed for the AGYW to increase the awareness and use of MCMs.

For example, one participant had this to share:

*… I think there is some level of knowledge and awareness among some AGYW on MCMs, especially those living near or in urban areas where more information on MCMs is available. I think more education and information on MCMs is required to increase the acceptance and use of MCMs and reduce the myths and misconceptions about MCMs in the streets*. (IDI, PT-03, 2023)

#### Prevention of Unintended Pregnancies

Many participants reported knowing the importance of using MCMs in the prevention of unintended pregnancies as the primary benefit of using modern contraceptives. They shared personal experiences on how MCMs have played a crucial role in helping young people and women avoid pregnancies they did not plan for. They further explained how MCMs have enabled them to pursue their dreams like continuing with school or doing business without fear of falling pregnant. Furthermore, they reported that the MCMs have given them a sense of security, allowing them to engage in sexual relationships without the constant worry of falling pregnant. For instance, one participant had this to narrate;

*“Using an MCM helps me take charge of my destiny and pursue what I want without worrying about falling pregnant. By using the MCM I can avoid unintended pregnancies and continue chasing my life goals without worrying”*. (IDI, PT-01, 2023).

Similarly, another participant added how MCMs have helped her to enjoy her sexual relationship without being worried about falling pregnant. She had this to say:

*“MCMs have helped me enjoy my sexual relationship with my boyfriend without fear of unwanted pregnancies; also I can continue to do business and travel to other districts to buy goods without worrying about falling pregnant…”.* (IDI, PT-03. 2023)

#### Empowerment and Control of Reproductive Choices

Many of the study participants reported how the use of MCMs has empowered them and enabled them to have control of their reproductive choices. They recounted that using MCMs gives them the power to choose when to have children or how many children they can have in a certain period. Some of them explained that they started to use the MCM after their first pregnancy so that the same challenges could not happen again. Some of the study participants reported that their first pregnancies were not planned and hence faced some difficulties during the time of delivery and taking care of their first child. They affirmed that now with the use of MCMs, they can choose the perfect time to have a child that they can take care of without any problems. For instance, one participant had this to share:

*My firstborn was not planned because I was not using MCMs, but now I am using an implant as a MCM; so* *for now I can choose when to have children and how many to have, for example, I will remove the implant when I am ready to have a second child.* (IDI, PT-05, 2023)

#### MCMs Use and Sexual Relationships

It was reported that using MCMs helped AGYW improve their sexual relationships. However, some of the participants reported using the MCMs secretly without the knowledge of their partners. They further explained that they use MCMs secretly because they were not sure how their partners would react if they informed them that they were using MCMs. Therefore, they have to keep it a secret to avoid arguments and breaking their relationship due to associating MCM use and having multiple sexual partners. Some of them acknowledged hearing their partners giving negative comments on the use of MCMs like women who use MCMs are prostitutes, so they know that they will not get their partners’ support on using MCMs and can ruin their relationships. For instance, one participant had this to say:

*There is no concern for my sexual relationship regarding the use of MCMs because I am using the contraceptive without the knowledge of my partner, I do not want him to know that I am using the MCM because I know he will not approve of it; and letting him know that I am using the method can ruin my sexual relationship.* (IDI, PT-07, 2023)

Another participant had this to share concerning her boyfriend’s opinion on MCMs use.

*“My boyfriend said that the modern contraceptive method that I am using will cause cancer to me and also I will fail to conceive and asked me to stop using it*. (IDI, PT-19, 2023)

#### Challenges and Side Effects

Many participants reported suffering from side effects arising from using MCMs. Some of the study participants further recounted experiencing some of the side effects related to the use of MCMs. The side effects that they suffered from included variation of the menstruation cycle and over-bleeding, irritation and regular urinary tract infection incidences after starting using MCMs. Furthermore, some of the participants reported to stop using MCMs after suffering from the side effects. For example, one participant had this to say:

*When I went to the health centre for the implant, they did not counsel me about any side effects that might arise from using the implant, in the beginning, my periods were irregular and I was over bleeding during my periods. I’m not sure if it is how it is supposed to be… I went back to the health facility for more help*. (IDI, PT-05, 2023).

One participant had this to share as to why she stopped using the MCM:

*When I started using the MCM, I experienced consecutive periods of extensive bleeding for several days. I didn’t know what to do because I was not informed of this kind of side effect when I got the MCM from the health facility. I was afraid; therefore, I went to the health facility and removed it…* (IDI, PT-06, 2023)

Another participant had this to say about the MCM’s side effects

*I was using the implant as a contraceptive method, but I started experiencing itchiness on my arm and regular urinary tract infections (UTIs). I was very afraid that the situation might get worse, so I went to the health facility to remove it. Nowadays I use condoms only because of what I went through with the implant.* (IDI, PT-08, 2023)

### Theme 2: Perception of AGYW on MCMs

Some participants expressed how information source can change their perception and attitudes on the use of MCM. Study participants revealed that they received some contradicting information regarding the use of MCMs which caused them to have concerns regarding the use of MCMs. Some of the issues reported by the study participants on their perception of MCMs included benefits and side effects. They further narrated that, they are scared when they hear myths and side effects that are related to the use of MCMs. The mentioned myths related to the use of MCMs are that they cause cancer and infertility. The study participants said that some youths stopped using the MCMs because of the myths attached to them. For instance, one participant had this to say:

*I have a lot of concerns about how I perceive the use of MCMs, for example when I first used an implant, I suffered from irregular periods and bleeding. If I combine those with what I heard people say in the streets about MCMs like causing cancer and infertility, I became very scared; and that is why I went to the health facility and had it removed; now I use the condom only, at least that does not have major side effects.* (IDI, PT-08, 2023)

Some participants reported how information sources influence their perceptions of using MCMs. They further explained how the information obtained from different sources can shape and change the whole course of deciding whether to use or not to use MCMs. Some of the study participants reported that when they received information from the health facilities regarding the MCMs and were assured that they were safe methods, that is when they decided to use them

One participant said;

*The big challenge is that some say the contraceptive methods bring health issues; they cause things like cancer, fibroids and infertility. However, the nurse who attended to me said that those are myths and misconceptions about the MCMs, and she assured me that the methods are safe and are used by many people. After receiving that explanation, I decided to use an implant as advised*. (IDI, PT-10, 2023)

#### Social Stigma and MCMs

Some participants reported how stigma and judgment have contributed to the low use of MCMs among AGYW. They recounted that the social stigma attached to MCMs use scare some youths away from health facilities to take MCMs. They further narrated that people in their community have the perception that if someone is using MCMs is a prostitute or has multiple sexual partners. This was demonstrated by one participant saying;

*It is hard to go to a health facility to take condoms or contraceptives, if people know that, then you will be labelled as a young prostitute or you have multiple sexual partners while it is not true, this labelling and stigma cause some young girls to fear to go to health centres to take MCMs.* (IDI PT-13, 2023)

#### Myths and Misconceptions on MCMs

Social influence was reported to influence MCMs’ perceptions and use among AGYW. They further reported that this influence often causes fear and negative attitudes toward the use of MCMs. They described how myths and misconceptions circulating within their social circles shaped their views on the use of MCMs. They further affirmed that family members and the broader community members have inaccurate information and myths on MCMs thus causing fear and confusion among AGYW on the use of MCMs. Among the numerous myths reported, included the belief that MCMs could cause diseases like cancer, fibroids, infertility and regular urinary tract infections (UTIs) and result in mentally disabled children. For instance, one participant said;

*People believe that if you use the MCMs you might be barren or get cancer, some people believe that if you use the MCMs you can get mentally disabled children that is what people say here regarding the use of MCMs*. (IDI, PT-11, 2023)

Another participant was concerned about the impact of oil in the condoms. She said:

*There are many myths regarding the use of MCMs here. I have heard some people saying that the oil in condoms causes HIV, while some people say that if you use the MCMs you will get cancer. So you end up being confused about MCMs.* (IDI, PT-01, 2023)

#### Cultural and Religious Beliefs

Cultural and religious beliefs were reported to affect AGYW’s perception of MCM use. Some of the study participants narrated that their religious teachings instilled the notion that using MCMs is a sin and could lead to health problems. Therefore, they are scared to use the MCMs because it is against their religion. Additionally, some participants reported on certain customs which emphasize the value of having many children, leading to fears that using MCMs might result in having fewer children or even cause barrenness. For example, one participant said;

*…My religious teachings do not allow MCMs use, as it is a sin but my religion allows the use of the natural family planning method, some religious preachers say MCMs can cause some diseases and interfere with the will of God on procreation. I do not want to go against my religion. So I use the natural family planning method*. (IDI PT-18, 2023)

Another participant had another view regarding having many children and she said:

*I do not know about the religious side of MCM use, but from the place where I come from having many children is considered a sign of wealth and social status, using modern contraceptives is neglected because it is believed to limit the number of children people can have. In my place, people have a strong belief that if you use MCMs, you might end up having very few children or none*. (IDI PT-14, 2023)

## DISCUSSION

This study explored the perceptions and experiences of AGYW regarding the use of modern contraceptive methods in the Malinyi District, Morogoro Region. Some participants were young and aged from 18 to 24 years. Educational-wise, many of them had completed secondary education, while few had attained post-secondary education. These findings differ slightly from previous studies which focused on younger participants with lower educational backgrounds.^29,30^ The differences may be attributed to differences in study settings and study participants.

### Experience in Using MCMs

In the case of knowledge and awareness, some of the participants demonstrated high knowledge of MCMs and their use. This is similar to findings from a study that reported on a fair understanding of young women regarding the use of MCM, benefits and side effects.^31^ However, in this study some participants reported having little knowledge of MCMs even though they were using them. This corroborates the findings from another study in Tanzaniawhich reported that some youths still lacked knowledge of MCMs which might lead to low usage.^31^ However, the current study contradicts the findings of Lwelamira et al. Lwelamira found, that 50% of those who were aware of MCMs had positive attitude on them. However, the study of Lwelamira et al^38^ included a wide range of women of reproductive age unlike the current study.^38^ The difference could be caused by the different study contexts.

More than half of the study participants emphasized the most common benefit of using modern contraceptives, which was the prevention of unintended pregnancies. This is similar to findings of a studies which were conducted in Kenya and Dodoma, which revealed that female college students tended to use MCMs when they believed such methods had positive benefits such as family planning, and preventing unwanted pregnancy and STDs.^21,32^ The benefit of preventing unintended pregnancies comes with other opportunities like education and career development. In the current study, many participants highlighted how using MCMs protected their education and careers. Additionally, some participants reported other empowering aspects, such as gaining control over their reproductive choices and attaining education and career opportunities, these findings align with previous studies conducted in Kenya that also underscored the advantages of fostering education and career development.^29^

The participants in this study shared their experiences of how MCMs enabled them to pursue education without the fear of being interrupted by unplanned pregnancies. Participants described completing their education at different levels and expressed gratitude to MCMs for giving them the freedom to focus on their academic goals and personal growth. This echoes the findings from a previous study that demonstrated how young women who had access to modern contraceptives were more likely to complete their secondary school education and have opportunities to continue pursuing higher education or careers.^21^

Moreover, the study findings reveal that some AGYW use MCMs secretly, fearing the reactions of their partners. Surprisingly, the secret use do not seem to negatively affect their relationships because their partners were unaware of it. Instead, it highlights the pressing need to educate men and boys about the importance and benefits of using modern contraceptives. Most men lack sufficient information about MCMs potentially leading to their discouragement of their partners from using them. This aligns with a previous study that emphasized the inadequate outreach to men regarding MCM usage.^33^ The current study reveals that AGYW often uses MCMs secretly without harming their relationships. These findings corroborate previous study findings conducted in Kenya Uganda and Tanzania.^35,36,37^ This calls for MCMs education that will include AGYW as well as their sexual partners, so that men are given MCMs education and allow their partners to use these methods freely.

This study also discovered that some narratives of the experienced challenges and side effects that study participants think may relate to MCM use such as itching irregular menstrual cycles, recurrent urinary tract infections and over-bleeding. These findings corroborate with a previous study conducted in Tanzania where participants also reported similar personal experiences of side effects like body changes, physiological effects, over-bleeding, irregular menstrual cycles and discomfort during sex.^37,38^ The similarity in findings between this study and the previous ones could be attributed to the common study setting in Tanzania and the shared experiences of AGYW in this region. Also, the current study findings on MCMs side effects corroborate with other study findings conducted in Madagascar, Kenya, and Malawi which revealed that women are not using MCMs due to some side effects they experience upon using them.^35,39,40^

Similarly, some participants in this study expressed experiencing stigma and judgment which instill fear of deciding to access and use MCMs. This is in agreement with the study findings in North West Tanzania, which reported that respondents perceived stigma as a barrier for young people accessing family planning.^31^ This implies that knowledge and awareness are not enough to make AGYW use MCMs because of some side effects experienced by some MCMs users. The side effects experienced if not well addressed can discourage some AGYW from using MCMs. MCMs interventions are needed for AGYW to address the side effects experienced and the measures to take when they experience the MCMs side effects.

### Perceptions of MCMs to AGYW

In this study, participants highlighted the impact of information sources on their perceptions and attitudes toward the utilization of modern contraceptive methods. Participants reported inconsistent information concerning MCMs, which caused fear and hesitations about using them. These findings are parallel with the study findings that were conducted in Madagascar, where it was reported that misinformation is among the barriers to contraceptive use among AGYW.^40^ Similarly, a study in Kenya reported that young women received inaccurate information which caused them not to use MCMs.^35^ Moreover, in the present study participants recognised the important role of information sources in shaping their decision-making processes regarding using MCMs. They revealed that information obtained from various sources significantly influenced their choices and altered their decisions regarding MCMs use. These current study findings are consistent with findings from studies conducted in Madagascar, Kenya and Malawi.^35,39,40^

Besides, some participants in this study revealed that their perceptions on MCMs use were influenced by cultural and religious beliefs. Furthermore, it was reported that religious teachings regarding MCMs lead to negative perceptions of the use of MCMs. Likewise, some tribes believe in the myth and misconceptions that using contraceptive methods will reduce the chances of having children since to their culture children are a source of wealth, so it is feared that using MCMs might limit the number of children in future. These findings align with a previous study conducted in India where youth held perceptions about MCMs side effects and being influenced by religious and cultural beliefs.^37^ This shows that more education on MCMs is needed to AGYW so that they understand the benefits of using MCMs.

Additionally, social influences and attitudes also played a role in perceived side effects, with some participants reporting that they heard about side effects from their peers or relatives who believed they were caused by MCMs. Those testimonies influenced the perceptions of MCM use among AGYW and contributed to fears and uncertainties regarding its use. These results were in agreement with findings from a study conducted in Kenya that highlighted the role of peer influence on young people’s contraceptive choices. Likewise, the same study reported that peers and other community members acted as the main sources of information regarding MCMs use, and their perceptions also heavily influenced the decision to use or not, also myths and misconceptions influence the use of MCMs.^35^

Equally, participants reported myths and misconceptions as other factors that influence perceived side effects. Participants further shared fear and concerns about contraceptive methods which mainly are due to myths and misconceptions for example, some participants explained that they have stopped using contraceptives due to fear of getting diseases like cancer or becoming infertile. The study findings match with the study findings conducted in Nigeria which revealed that the main barriers to MCM uptake among young women were myths and misconceptions.^41^ This shows that more education on MCMs is needed for AGYW so as to address the myths and misconceptions surrounding MCM use.

Difficulties in accessing reproductive health information, stigma and physical punishments have been shown to constitute hindrances to the use of modern contraceptive methods (MCMs) among AGYW as revealed in a study by Nyblade, et al. conducted in East Africa.^31^ The study further discovered that female and male adolescent youths aged between 11 and 26 years in Tanzania experienced challenges in accessing reproductive health information from parents and teachers. However, the study was limited only to North-West Tanzania which makes it hard to be generalized, but gives some insight into the MCMs dynamics. Another study conducted by Lwelamira, et al.^38^ on knowledge, attitude and perceptions of using MCMs among married women aged between 15 and 49 years in the Mpwapwa district revealed knowledge of modern contraceptives in a study population to be high and a substantial proportion of respondents had positive attitude towards MCMs. Half (50%) of respondents were aware of modern contraceptives and thought that the benefits of modern contraceptives outweighed the negative effects; and 42% agreed that they could recommend the use of modern contraceptives to a friend. However, despite the presence of positive attitudes towards modern contraceptives by a good number of women in the study population, negative attitudes of husbands towards modern contraceptives can be one of the obstacles to the success of campaigns to increase MCM use in the study area.^38^ This imply that negative attitude towards MCMs use might deter some AGYW from using them. More education and awareness is needed to AGYW on the benefits of using MCMs.

Apart from that Ministry of Health, Gender, the Elderly and Children, reported that younger unmarried women aged 15–19 years were perceived to be less likely to use condoms during sexual intercourse than unmarried young men aged 20–24 years ^20^. Similarly, Kara et al.^32^ show that female college students in Tanzania choose not to use MCMs due to religious beliefs.

This might imply that perceptions of people, social norms on MCMs, accessibility of MCMs, information, knowledge, myths and peer influence might affect the utilization of the MCMs regardless of being aware of the importance and benefits of using MCMs. This calls for more intervention on raising awareness and imparting more knowledge to AGYW on the importance of using MCMs.

### Study Limitation and Mitigation

The current study might have faced some social desirability bias where participants were providing responses that they believed were socially acceptable or expected, rather than sharing their true feelings and experiences regarding MCM use. This limitation was mitigated by providing clear information about the objectives of the study and requesting the study participants to be honest. Furthermore, only AGYW who were using MCMs or had previously used them were selected to participate in this study hence excluding others who had not or were not using MCMs. Also, men were excluded from this study.

Moreover, AGYW were initially hesitant to participate in the study and discuss sensitive topics related to sexual health due to stigma or cultural taboos surrounding contraception and sexual behaviour. However, this was managed by assuring them of confidentiality and letting them select the place where they would prefer interviews to be conducted avoiding being labelled as prostitutes.

## CONCLUSION

The study revealed that some AGYW had knowledge and awareness of MCMs. Additionally, the study findings revealed that some participants use MCMs in the prevention of unintended pregnancies, giving them empowerment and control over their reproductive choices, and enhancing their sexual relationships. Some participants reported using the MCMs without the knowledge of their partners. Furthermore, the participants reported the challenges they face in using the MCMs and the perceived side effects they suffer from using MCMs. Similarly, social stigma and judgment, information sources on MCMs, social influence and attitudes, cultural and religious beliefs, myths and misconceptions were reported barriers to MCM. We recommend that healthcare workers should continue giving education on MCMs and counsel AGYW on MCMs. MCMs’ education and awareness should be extended to their partners as well.
